# Biomimetic Nanoplatform for Targeted Glioblastoma Therapy via Concurrently Triggering GPX4/DHODH Mediated Ferroptosis

**DOI:** 10.1002/advs.202521236

**Published:** 2026-03-23

**Authors:** Guodong Ren, Xuewei Wang, Panpan Li, Hao Wu, Zhifei Bi, Qi Gao, Bolong Ma, Zixuan Tian, Jianfei Liu, Fuqiang Feng, Wen Liu, Lin Li, Chengwu Zhang

**Affiliations:** ^1^ School of Basic Medical Sciences Shanxi Medical University Taiyuan China; ^2^ Shanxi Province Brain Degenerative Diseases Precision Diagnosis and Treatment Engineering Research Center Shanxi Medical University Jinzhong China; ^3^ State Key Laboratory of Flexible Electronics (LoFE) & Institute of Flexible Electronics (IFE) Northwestern Polytechnical University Xi'an China; ^4^ Department of Neurosurgery, Shanxi Province Cancer Hospital/Shanxi Hospital Affiliated to Cancer Hospital Chinese Academy of Medical Sciences/Cancer Hospital Affiliated to Shanxi Medical University Taiyuan China; ^5^ State Key Laboratory of Flexible Electronics (LoFE) & Institute of Flexible Electronics (IFE) Xiamen University Xiamen China

**Keywords:** DHODH, ferroptosis, glioblastoma, GPX4, H‐MnO_2_

## Abstract

Glioblastoma multiforme (GBM), the most aggressive and lethal type of brain cancer, is a considerable threat to human health. Conventional therapeutic modalities fail to yield satisfactory outcomes; therefore, a more effective intervention strategy is urgently required. Ferroptosis, a novel type of cell death, has potential for GBM therapy. However, its efficacy is substantially compromised by the tumor‐intrinsic anti‐ferroptosis defense system. Moreover, approaches that trigger ferroptosis by modulating a single target are insufficient. Thus, it is imperative to simultaneously inhibit anti‐ferroptotic regulators to overcome compensatory pathways and achieve robust tumor eradication. Therefore, in the present study, a multifunctional nanoplatform, hollow mesoporous manganese dioxide (H‐MnO_2_)‐hemin‐leflunomide@membrane (**MHL@M**), is proposed and fabricated. GBM cell membrane coating enables blood‐brain barrier (BBB) penetrating and tumor‐targeting properties. H‐MnO_2_ consumes the overexpressed GSH in the tumor microenvironment (TME), and as derived Mn^2+^ converts H_2_O_2_ into more toxic •OH, resulting in a chemodynamic therapy (CDT) effect. Hemin downregulates glutathione peroxidase 4 (GPX4), and leflunomide inhibits dihydroorotate dehydrogenase (DHODH), which synergistically triggers ferroptosis. Both in vitro and in vivo results demonstrate that **MHL@M** has excellent tumor‐targeting, TME‐responsive, and ferroptosis activation capacities. This study provides a solid foundation for the development of ferroptosis‐based therapeutic strategies for GBM.

## Graphical abstract

Considering the great challenge in Glioblastoma multiforme (GBM) treatment, the authors designed and synthesized a multifunctional nanoplatform that effectively induced ferroptosis by synergetically inhibiting intrinsic intrisinc anti‐ferroptosis proteins, GPX4 & DHODH, and realized excellent in vitro and in vivo anti‐GBM effect.

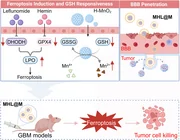

## 1 Introduction

Glioblastoma multiforme (GBM), one of the most aggressive intracranial tumors, is characterized by poor prognosis, and high recurrence rates [1]. Currently, the standard care for GBM is surgical resection, followed by adjuvant radiotherapy and concurrent chemotherapy [2]. However, complete surgical resection remains unattainable because of the extensive infiltration of tumor cells into the adjacent normal brain parenchyma [3]. Over the past few decades, temozolomide (TMZ) has been the first‐line therapeutic agent for GBM. However, its efficacy is notably undermined in a subset of patients with GBM, which is attributed to innate drug resistance [4, 5]. Drugs, such as sorafenib and sunitinib, show promising activity in preclinical studies, but they fail to provide significant clinical benefits for patients with GBM partially due to their limited ability to cross the blood‐brain barrier (BBB) [6]. Radiation therapy is also limited by its narrow therapeautic windows; low‐dose radiation cannot produce effective therapeutic effects, and high‐dose radiation might induce toxicity in normal tissues and organs [7]. Therefore, more precise and efficacious therapeutic strategies for GBM are in high demand but are unmet.

The substantial potential of ferroptosis‐based strategies in tumor therapy has garnered attention [8, 9, 10]. Ferroptosis is an iron‐dependent form of regulated cell death mediated by lipid peroxidation, which is distinct from apoptosis, necrosis, necroptosis and pyroptosis [11]. Given the lipid enrichment and iron deposition of the brain, ferroptosis is a promising strategy for GBM treatment [12, 13]. However, ferroptosis is negatively regulated by glutathione peroxidase 4 (GPX4), localized in the cytosol and mitochondria, as well as dihydroorotate dehydrogenase (DHODH), an iron‐containing flavin‐dependent enzyme localized to the inner mitochondrial membrane [14, 15]. In GBM, GPX4 functions as a central regulator of ferroptosis by reducing toxic lipid hydroperoxides to non‐toxic lipid alcohols [16]. The expression level of GPX4 was up‐regulated in glioma patients [17]. Meanwhile, overexpression of GPX4 promoted ferroptosis resistance in GBM [18]. Genetic depletion or pharmacological inhibition of GPX4 could induce GBM cells to undergo ferroptosis [19]. Zhang et al. prepared FeOOH nanoshuttles that achieved cancer inhibition by regulating the SLC7A11/GSH/GPX4 axis [20]. Similarly, Liu et al. designed an extracellular vesicle‐delivered CRISPR/Cas9 for GBM radiosensitization therapy with inactivation of GPX4 [21]. However, inhibiting GPX4 alone often fails to achieve complete tumor eradication due to compensatory resistance pathways. DHODH, required for *de novo* pyrimidine synthesis, potently suppresses ferroptosis through a mechanism independent of GPX4. Mechanistically, DHODH reduced ubiquinone to ubiquinol, thereby providing an alternative mechanism for inhibiting mitochondrial lipid peroxidation that was distinct from the canonical cytosolic GPX4 pathway [22]. DHODH was elevated in GBM, which was closely related to proliferation and drug‐resistance of GBM cells [23]. The inhibition of DHODH also exhibited excellent efficacy in inducing ferroptosis, effectively suppressing the proliferation of cancer cells. Yin et al. developed an engineered metal‐phenolic networks (TBFP‐MT MPNs) by disrupting the DHODH defense systems, to suppress resistant GBM growth [24]. Although solely inhibiting GPX4 or DHODH displayed anti‐tumor effects, the outcome is unsatisfactory. It is found that there is a compensatory interplay between the above ferroptosis defensive axes. Mao et al. found that, when GPX4 expression was inhibited, metabolic flux could be restored via the upregulation of DHODH activity, thereby preserving mitochondrial integrity and compensating for the loss of cytosolic protection [25]. This indicated that the GPX4 and DHODH pathways function as parallel and complementary ferroptosis suppression systems in GBM cells. Consequently, a dual‐targeting strategy designed to simultaneously disrupt both the cytosolic (GPX4) and mitochondrial (DHODH) antioxidant systems has potential for overcoming compensatory resistance and maximizing ferroptosis induction in GBM.

The BBB is another major barrier to GBM treatment that severely hinders the penetration of therapeutic agents [26, 27]. To overcome this challenge, some novel modification strategies are developed, including transferrin [28], liposomes [29] and cell membranes [30]. Among these, GBM‐derived cell membrane (CM)‐coating exhibits exceptional advantages owing to their inherent tumor‐targeting capacities and biomimetic properties [31, 32, 33]. Wang et al. successfully developed a biomimetic hypoxia‐triggered RNAi nanomedicine (poly (MIs)/PTX@PEI/siPGK1@CCM) that could efficiently penetrate the BBB and specifically accumulate at GBM sites [34]. In addition, a responsive release can also enhance the precise treatment effect of drugs [35]. Manganese dioxide (MnO_2_) was a tumor microenvironment (TME) responsive mineral. It could be reduced to manganese ions (Mn^2+^) by glutathione (GSH) within the TME, releasing the encapsulated therapeutic agents. Mn^2+^ could catalyze the H_2_O_2_ to generate toxic hydroxyl radicals (•OH) to achieve chemodynamic therapy (CDT) [36, 37, 38].

**SCHEME 1:**
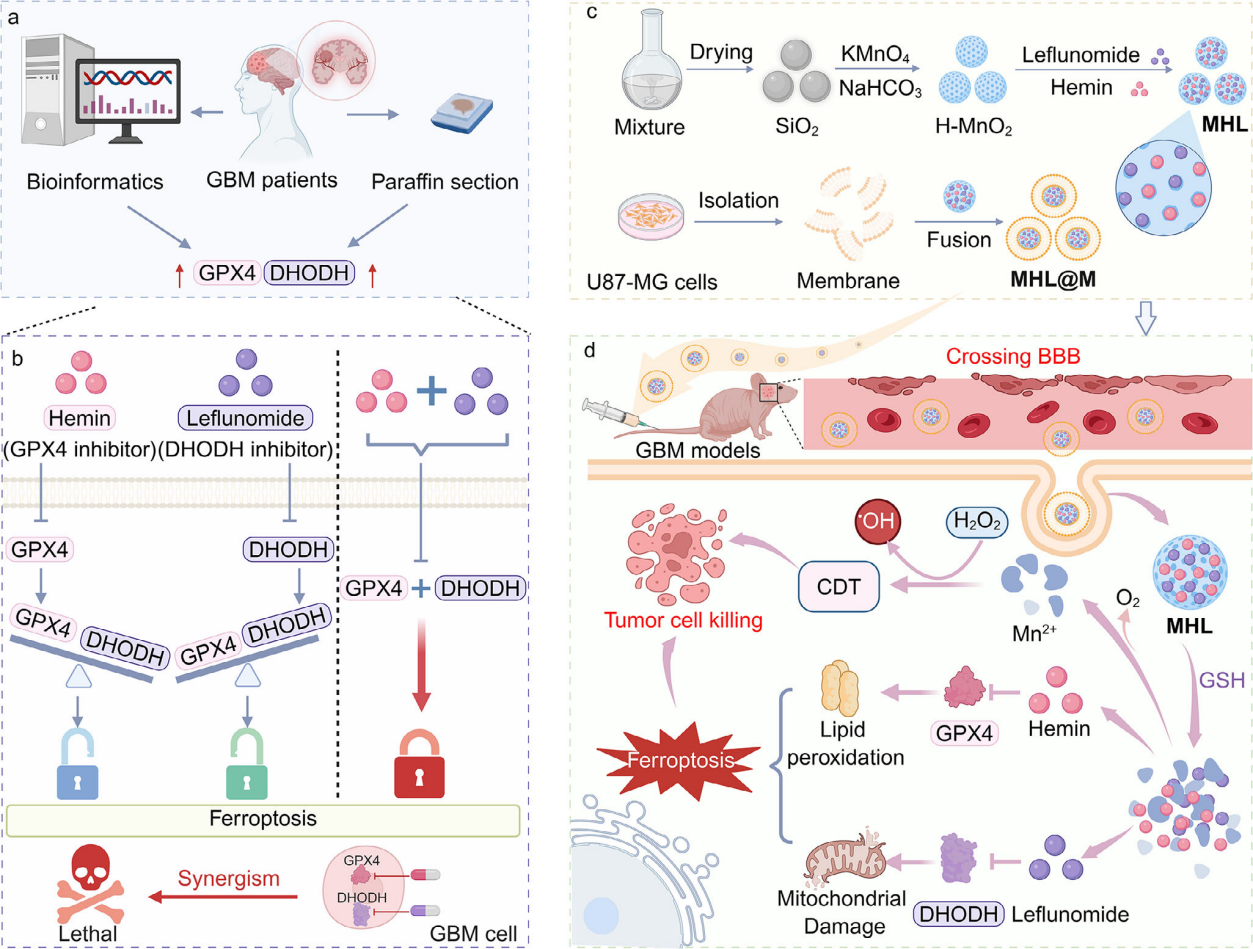
Schematic illustration of the preparation and treatment mechanism of the MHL@M. a) Bioinformatics screening of anti‐ferroptosis genes in patients with GBM. b) Compensatory response of GPX4/DHODH inhibition. c) Synthesis scheme of **MHL@M**. d) Anti‐tumor mechanism of **MHL@M**.

With the aforementioned issues in mind, a biomimetic multifunctional nanoplatform hollow mesoporous manganese dioxide (H‐MnO_2_)‐hemin‐leflunomide@membrane (**MHL@M)**, composed of H‐MnO_2_, hemin, leflunomide and CM, was developed to achieve a synergistic treatment of GBM. The CM endowed **MHL@M** with BBB penetration and GBM homologous targeting capacities, thereby enhancing its concentration within tumor cells/tissues. As a responsive module, H‐MnO_2_ could be reduced to Mn^2+^ by depleting the overexpressed GSH, downregulating the GPX4 and activating the CDT. Hemin further reduced GPX4 expression and provided ferrous ions (Fe^2+^) to trigger ferroptosis. Leflunomide, a specific DHODH inhibitor, enhanced the ferroptosis response. Experimental results showed that **MHL@M** achieved excellent GBM therapeutic effect via efficiently activating ferroptosis (Scheme 1). The innovative design of the biomimetic nanoplatform contributed to the development of a clinical intervention strategy.

## 2 Results and Discussion

### 2.1 Overexpression of GPX4 and DHODH in GBM

**FIGURE 1:**
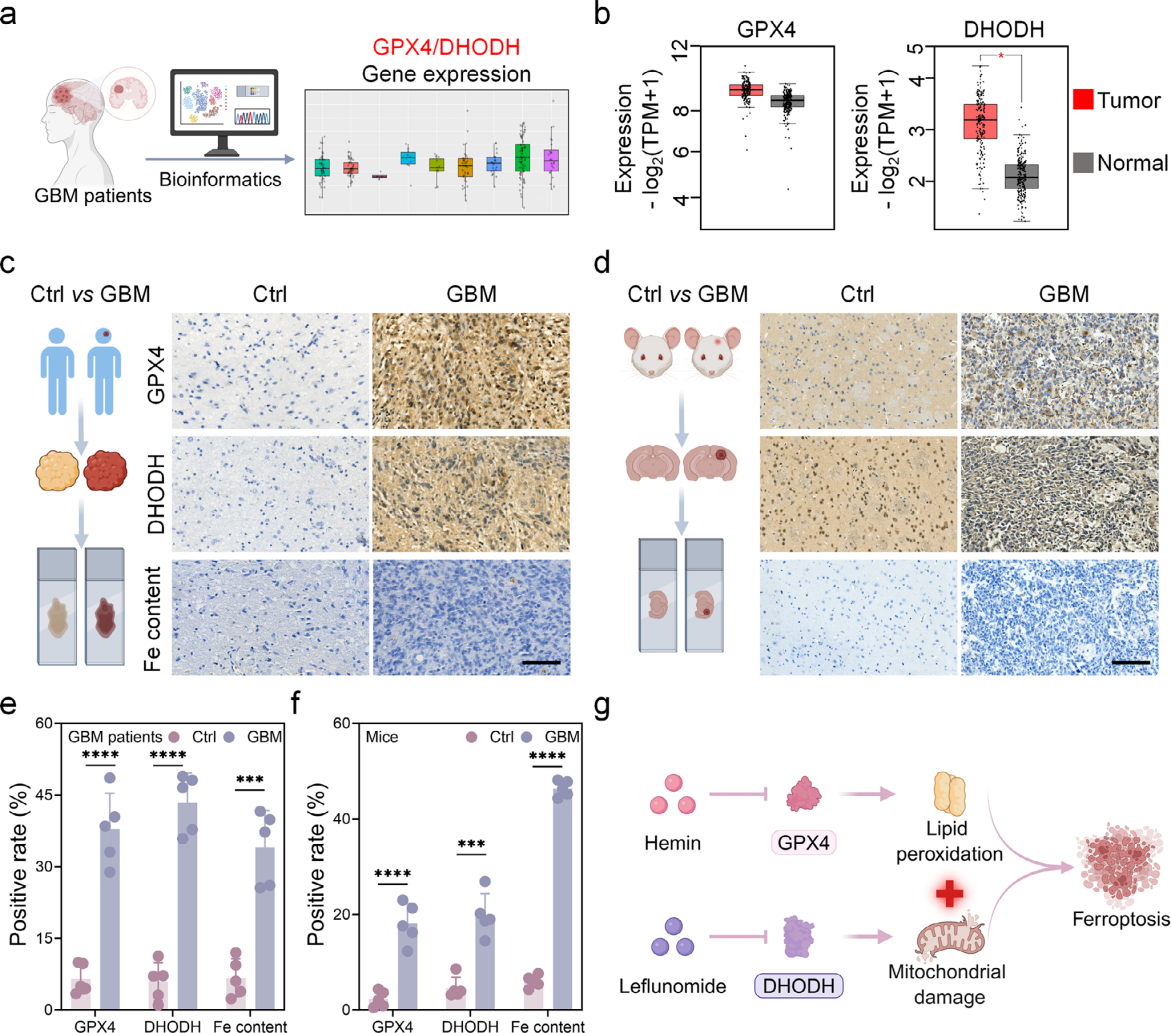
Expression profiles of GPX4 and DHODH in GBM. a) Schematic illustration of the bioinformatics analysis, and the gene expression detail was shown in supporting information (Figures S1, S2). b) Expression analysis of GPX4 and DHODH in GBM using the GEPIA database. c) IHC staining images of GPX4 and DHODH and prussian blue staining and e) their quantitative analysis in clinical GBM specimens and adjacent non‐neoplastic brain tissues (*n* = 5, scale bar = 100 µm). d) IHC staining images of GPX4 and DHODH expression and prussian blue staining and f) their quantitative analysis in brain tissues from control mice and orthotopic GBM models (*n* = 5, scale bar = 100 µm). g) Schematic illustration of the concurrent inhibition of GPX4 and DHODH.

The therapeutic efficacy of ferroptosis‐based strategies in GBM are often compromised. GPX4 and DHODH are key antioxidants that play crucial roles in ferroptosis [39]. To verify the expression profile of GPX4 and DHODH in GBM, bioinformatics analysis and GBM tissue immunohistochemical (IHC) staining were performed. Using UALCAN and GEPIA databases [40, 41], we analyzed the mRNA expression levels of GPX4 and DHODH in GBM and normal brain tissues. As shown in Figure 1a, b and Figures S1, S2, the expression of GPX4 and DHODH in GBM tissues was significantly higher than that in normal tissues. GPX4 expression levels positively correlated with glioma malignancy and negatively correlated with patient survival time, with higher levels of GPX4 indicating a worse prognosis using Chinese Glioma Genome Atlas (CGGA) database (Figure S3) [42]. To further verify the bioinformatics findings, IHC staining was performed on human GBM specimens and orthotopic GBM mice brain tissues. Consistently, the expression of GPX4 and DHODH was markedly elevated in specimens from patients with GBM compared with that in adjacent non‐neoplastic brain tissues (Figure 1c, e). In brain tissues of orthotopic GBM mice models, significantly elevated GPX4 and DHODH were also observed (Figure 1d, f). Therefore, it was confirmed that concurrently inhibiting both GPX4 and DHODH using nanoplatform could serve as an alternative strategy for the effective treatment of GBM (Figure 1g). Notably, the Fe content was clearly elevated within the brain of GBM samples compared with that in normal samples, indicating the potential of ferroptosis in GBM treatments (Figure 1c–f). Collectively, simultaneous suppression of GPX4 and DHODH held potential to induce “explosive” ferroptosis.

### 2.2 Preparation and Characterization of MHL@M

**FIGURE 2:**
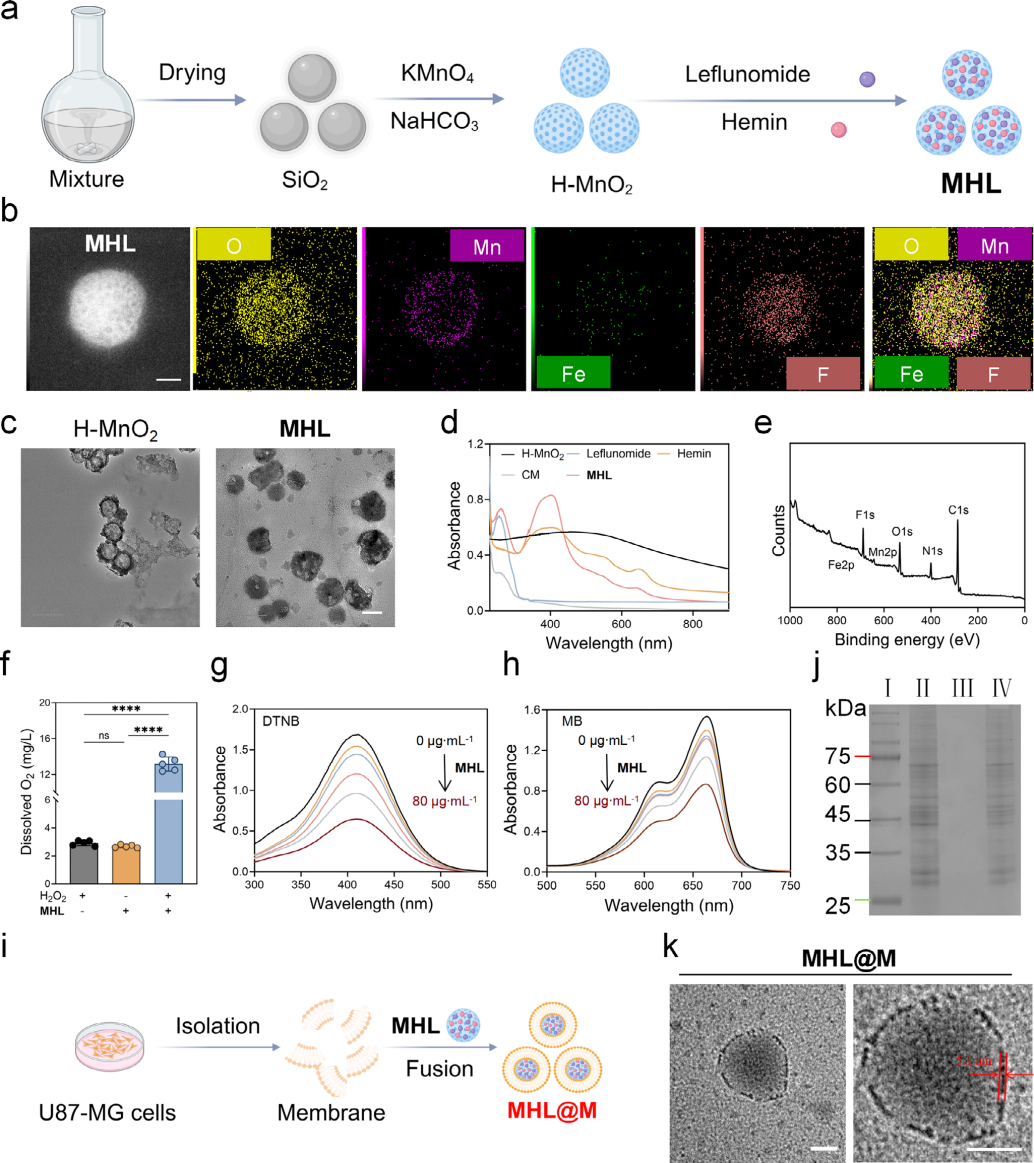
Characterization of MHL@M. a) Schematic illustration of the fabrication of **MHL**. b) Mapping results of **MHL** (scale bar = 25 nm). c) TEM images of H‐MnO_2_ and **MHL** (scale bar = 100 nm). d) Absorbance spectra of different units. e) XPS spectra of **MHL**. f) The level of O_2_ in various groups. g) GSH depletion capacity of **MHL** detected by DTNB probe (50 µM). h) CDT capacity of **MHL,** and MB (0.15 mM) served as indicator. i) Schematic illustration of the process for encapsulation of CM onto **MHL**. j) SDS‐PAGE protein analysis of marker (I), U87‐MG membrane (II), **MHL** (III) and **MHL@M** (IV). k) TEM images of **MHL@M** (scale bar = 100 nm).

Given the anti‐ferroptotic role and expression profile of GPX4 and DHODH in GBM, a multifunctional nanoplatform (**MHL@M**) composed of H‐MnO_2_, hemin (GPX4 inhibitor), leflunomide (DHODH inhibitor) and CM, was designed and fabricated to treat GBM by activating facilitated ferroptosis. As shown in Figure 2a, H‐MnO_2_ was fabricated using the hard template method, and then loaded with hemin and leflunomide to obtain MnO_2_‐hemin‐leflunomide (**MHL**). After obtaining the **MHL**, the loading contents of hemin and leflunomide were determined using UV–vis absorption spectroscopy. As shown in Figure S4, loading contents of hemin and leflunomide were 23.08% and 32.14%, respectively. Elemental mapping revealed the presence of manganese (Mn), iron (Fe), and fluorine (F), confirming the successful loading of hemin and leflunomide onto H‐MnO_2_ (Figure 2b). Subsequently, physicochemical properties of **MHL** were assessed. Transmission electron microscopy (TEM) images of H‐MnO_2_ and **MHL** showed that they were spherical with average sizes of approximately 91.50 and 103.50 nm, respectively (Figure 2c). The absorption spectrum revealed that the characteristic absorption peaks of H‐MnO_2_, hemin and leflunomide were all presented in **MHL** (Figure 2d). X‐ray photoelectron spectroscopy (XPS) and Fourier‐transform infrared spectroscopy (FTIR) confirmed the successful preparation of **MHL** (Figure 2e; Figures S5, S6).

Hypoxia and excess GSH are distinctive hallmarks of the TME, which severely hinder the therapy for GBM [43, 44]. H‐MnO_2_ had excellent catalase and GSH‐depletion capacities, indicating its substantial potential in GBM intervention [45]. To assess the TME‐responsive drug release capability of the nanoplatform, the simultaneous release behaviors of hemin, leflunomide, and Mn^2+^ from **MHL** were evaluated. Under high GSH conditions (mimicking the TME), the three components exhibited triggered release, with cumulative release reaching approximately 55.10%, 63.00%, and 87.49% within 24 h, respectively. These results confirmed the excellent TME‐triggered release behavior of **MHL**, which provided a solid foundation for precise drug delivery to GBM (Figure S7). As shown in Figure 2f, **MHL** exhibited excellent oxygen (O_2_) generation performance. Meanwhile, changes in the morphology, and zeta potential of **MHL** incubated with GSH indicated that **MHL** was sensitive to GSH (Figure S8a, b). Additionally, the results of the 5,5'‐dithiobis‐(2‐nitrobenzoic acid) (DTNB) assay showed that the GSH‐depletion capacity of **MHL** was in a concentration‐dependent manner (Figure 2g). Moreover, in the presence of GSH, H‐MnO_2_ was reduced to Mn^2+^, which could serve as a CDT agent. To assess chemodynamic properties of the nanoplatform, methylene blue (MB) was used to detect •OH radicals. As shown in Figure 2h, after incubation with GSH, **MHL** catalyzed H_2_O_2_ to generate •OH in a dose‐dependent manner. These results indicated that **MHL** had excellent TME‐responsive and O_2_/•OH generation capacities.

Moreover, to enhance the BBB crossing and overcome off‐targeting toxicity of traditional treatments, **MHL** was encapsulated in CM, forming **MHL@M** (Figure 2i) [46]. The absorption spectrum confirmed the successful fabrication of **MHL@M** (Figure S9). The protein components of **MHL@M** were examined using sodium dodecyl sulfate‐polyacrylamide gel electrophoresis (SDS‐PAGE). Similar protein bands in the U87‐MG cell membranes and **MHL@M** indicated good retention of CM proteins on the biomimetic nanoparticles (Figure 2j). Moreover, cell surface glycoprotein CD44, one specific cell membrane protein, was detected with western blotting assay. As shown in Figure S10, bands corresponding to CD44 were observed in **MHL@M** and the positive control group, but not in the **MHL** group. Above results indicated the successful preparation of membrane coated **MHL**. TEM images indicated that the thickness of CM was 5.1 nm (Figure 2k). In addition, changes in the hydrodynamic diameter and zeta potential were also indicated the successful fabrication of **MHL@M** (Figure S11a, b). Notably, hemolysis experiments indicated that **MHL@M** exhibited good biosafety, guaranteeing its further biological applications (Figure S12).

### 2.3 GBM‐Targeting and BBB Penetrating Performance of MHL@M

**FIGURE 3:**
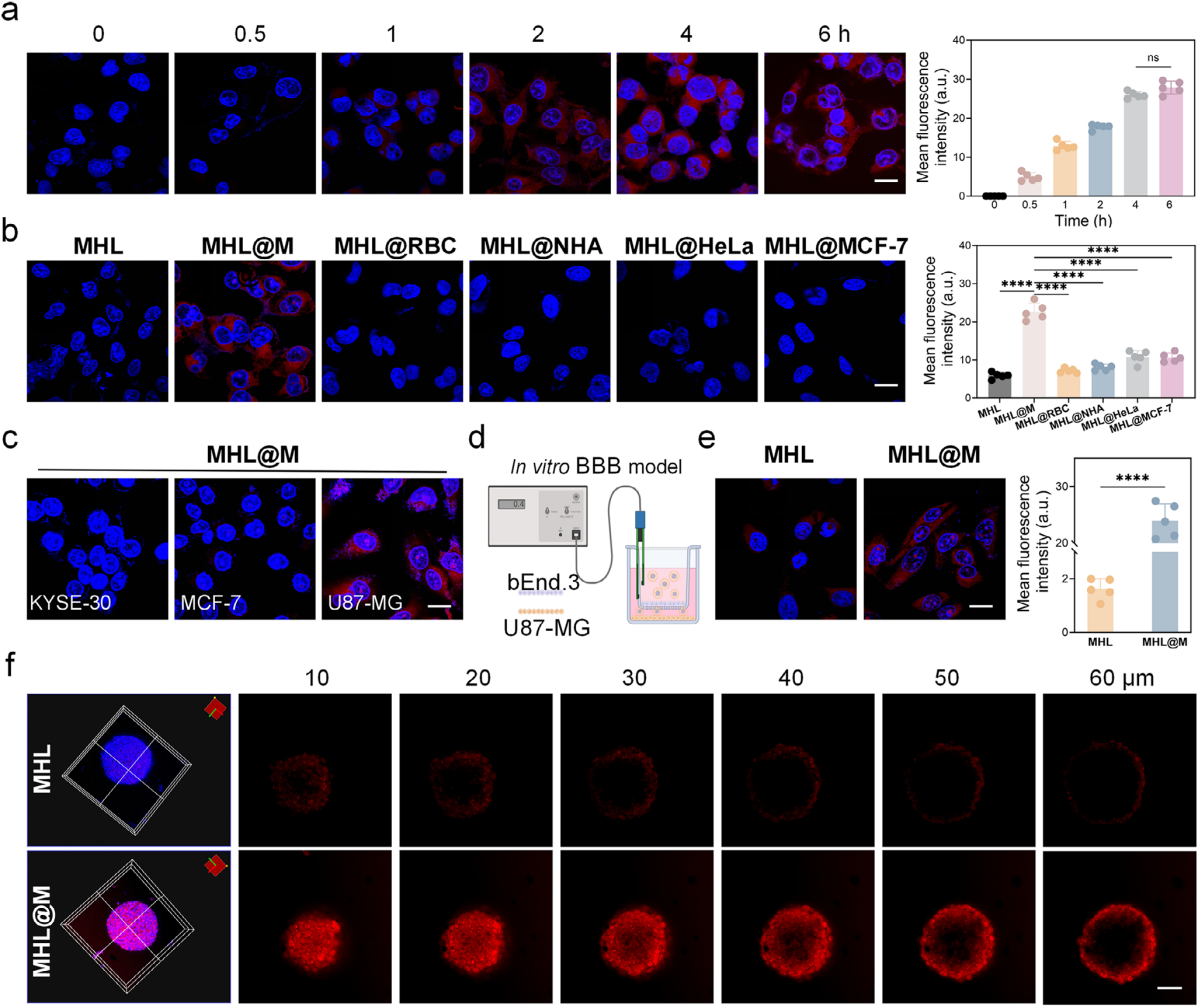
Cellular uptake and BBB penetration performance of MHL@M. a) CLSM of U87‐MG cells incubated with **MHL@M** (100 µg∙mL^−1^) for different time (*n* = 5, scale bar = 20 µm). b) CLSM images of U87‐MG cells incubated for 4 h with 100 µg∙mL^−1^ of different materials (**MHL**, **MHL@M**, **MHL@RBC**, **MHL@NHA**, **MHL@HeLa**, and **MHL@MCF‐7**, *n* = 5, scale bar = 20 µm). c) CLSM images of different cells (KYSE‐30, MCF‐7 and U87‐MG) incubated with **MHL@M** (100 µg∙mL^−1^) for 4 h (*n* = 5, scale bar = 20 µm). d) Schematic illustration of the in vitro BBB model (Transwell). e) CLSM of U87‐MG cells incubated for 4 h with 100 µg∙mL^−1^ of **MHL** or **MHL@M** in vitro BBB model (*n* = 5, scale bar = 20 µm). f) Tumor penetration capacity of **MHL** and **MHL@M** in U87‐MG cell spheroids (*n* = 5, scale bar = 100 µm).

Next, the performance of **MHL@M** was assessed at the cellular level. We examined whether **MHL@M** could be efficiently taken up by GBM cells. Confocal laser scanning microscopy (CLSM) images and flow cytometry analysis results showed that **MHL@M** was efficiently internalized by U87‐MG cells and reached a steady state within 4 h (Figure 3a; Figure S13a). To further verify its targeting specificity, U87‐MG cells were treated with **MHL** coated with membranes derived from different cell lines, including red blood cells (RBC), human astrocytes (NHA), human cervical carcinoma cells (HeLa) and human breast cancer (MCF‐7). As shown in Figure 3b and Figure S13b, U87‐MG cells showed a more efficient uptake of **MHL@M** than that in other membrane‐coated **MHL**. Human esophageal cells (KYSE‐30), MCF‐7 and U87‐MG cells were used to further assess the parental cell‐specific homing preference of **MHL@M**. As shown in Figure 3c and Figure S13c, d, **MHL@M** exhibited the best uptake performance by U87‐MG cells, demonstrating the U87‐specific uptake preference of **MHL@M**.

To further verify the BBB penetration capacity of **MHL@M**, we established an in vitro BBB model with mouse brain microvascular endothelial cells (bEnd.3 cells) and verified it using transendothelial electrical resistance (TEER, Figure 3d). As shown in Figure S14, TEER of the bEnd.3 cell layer reached 200 Ω∙cm^2^, indicating successful formation of the BBB model. BBB penetration assays demonstrated the strong capacity of **MHL@M** to cross the BBB and subsequently target GBM cells (Figure 3e). GBM is a multiple layer's cell spatial structure; therefore, it was necessary to investigate whether **MHL@M** could penetrate the tumor tissue. To assess this property, we established 3D cell sphere culture model using U87‐MG cells. As shown in Figure 3f, compared with **MHL**, **MHL@M** showed deeper penetration into the 3D cell sphere, as evidenced by the stronger fluorescence signal. These results demonstrated that **MHL@M** possessed excellent tumor‐targeting, and BBB and tumor tissue penetration capacities.

### 2.4 In Vitro Anti‐Tumor Performance of MHL@M

**FIGURE 4:**
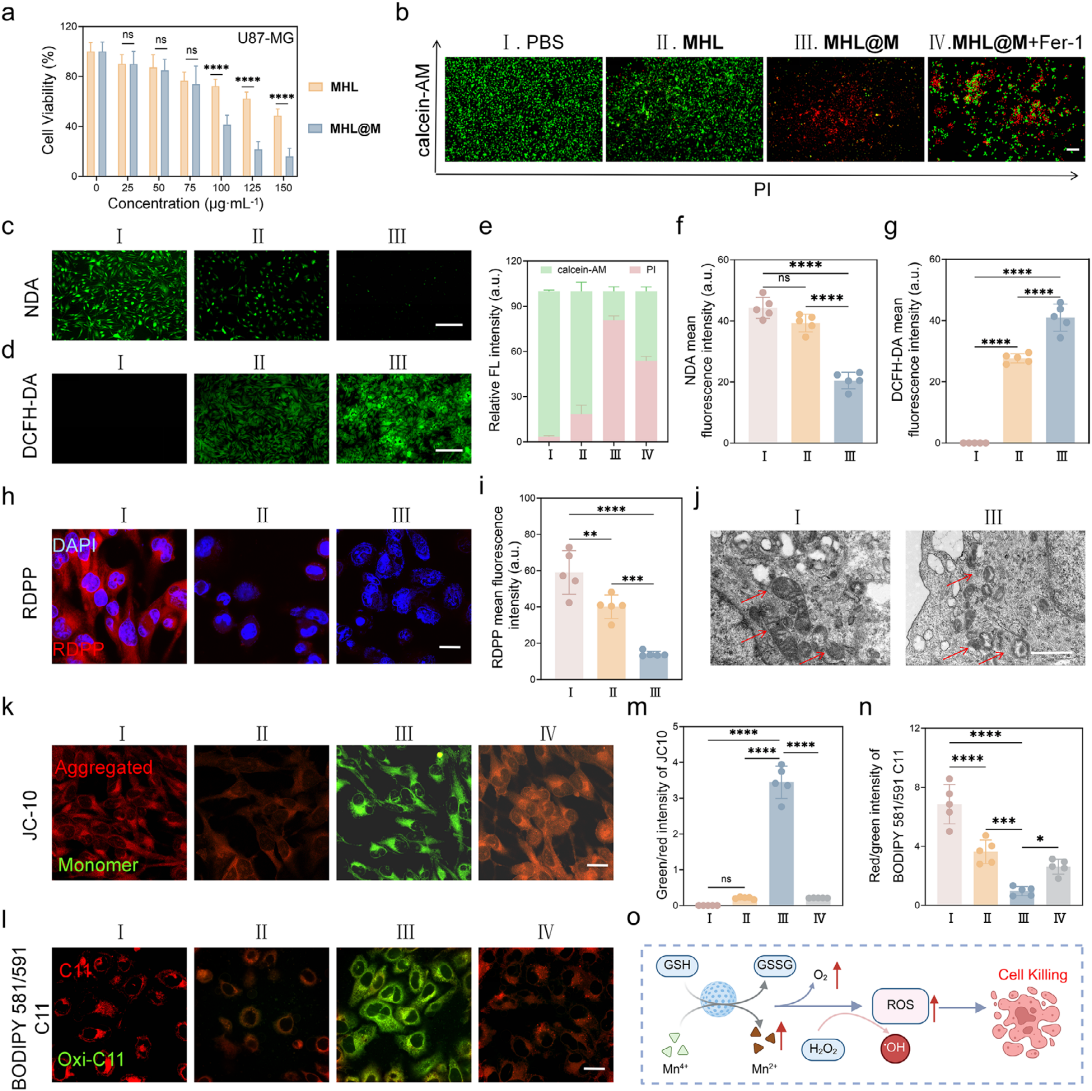
In vitro anti‐tumor performance of MHL@M. a) MTT assay of **MHL@M**’s cell‐killing capacity. b) Images and e) qualitative analysis of U87‐MG cells stained with calcein‐AM/PI after treating with different drugs for 24 h (*n* = 5, scale bar = 200 µm). c) Fluorescence imaging and f) quantitative analysis of U87‐MG cells incubated with **MHL** and **MHL@M** using GSH probe (*n* = 5, scale bar = 200 µm). d) Fluorescence imaging and g) quantitative analysis of U87‐MG cells incubated with **MHL** and **MHL@M** using ROS probe (*n* = 5, scale bar = 200 µm). h, i) O_2_‐generation capacity of **MHL@M** at the cellular level (*n* = 5, scale bar = 20 µm). j) Bio‐TEM results of U87‐MG cells in PBS and **MHL@M** (100 µg∙mL^−1^) group (scale bar = 1 µm). k) CLSM imaging and m) qualitative analysis of MMP in U87‐MG cells with different treatments (*n* = 5, scale bar = 20 µm). l) CLSM imaging and n) qualitative analysis of LPO in U87‐MG cells treated with different drugs followed by probe staining. (*n* = 5, scale bar = 20 µm). o) Schematic illustration of the therapeutic mechanism of **MHL@M**. (I: PBS, II: **MHL**, III: **MHL@M**, IV: **MHL@M**+Fer‐1).

Triggering its uptake behavior in U87‐MG cells, we further tested the cell‐killing capacity of **MHL@M** via methyl thiazolyl tetrazolium (MTT) and calcein AM/PI experiments. As shown in Figure 4a, b, e, **MHL@M** exhibited the most prominent cell‐killing effect, while Fer‐1 partially reversed this observation. The viability of the NHA cells remained above 80% after incubation with **MHL** and **MHL@M**, indicating their good biocompatibility (Figure S15). The MTT assay results revealed that the cell‐killing of free hemin and leflunomide was significantly lower than that of the **MHL@M** group. This suggested that the MHL@M nanoplatform achieved superior anti‐tumor efficacy compared to the respective monotherapies (Figure S16). U87‐MG cells subjected to **MHL@M** showed the lowest number of colony units (Figure S17). Invasion and metastasis are notorious features of GBM, therefore, to validate the anti‐invasion and anti‐metastatic potential of **MHL@M**, wound healing and transwell experiments were performed. As shown in Figure S18, **MHL@M** exhibited excellent anti‐invasion and anti‐metastatic properties. The intracellular GSH concentration was investigated using a naphthalene‐2,3‐dicarboxaldehyde (NDA) probe; **MHL@M** exhibited an outstanding GSH‐depleting capacity (Figure 4c, f; Figure S19a–d). The intracellular ROS generation capacity of **MHL@M** was evaluated using the commercially available ROS probe, 2,7‐dichlorodihydrofluorescein diacetate (DCFH‐DA). As illustrated in Figure 4d, g, and Figure S19e, the **MHL@M** group exhibited the strongest fluorescence signal. Furthermore, the changes in fluorescence intensity of [Ru(dpp)_3_]Cl_2_ (RDPP), a commercial oxygen probe, indicated the O_2_‐generation capacity of **MHL@M** (Figure 4h, i). Collectively, these results showed **MHL@M** had excellent tumor targeting, GSH‐depletion and O_2_/ROS generation capacities.

Inhibition of GPX4/DHODH and triggering threlease of ROS could induce mitochondrial dysfunction and subsequent ferroptosis [18, 22] To verify the performance of **MHL@M** in activating ferroptosis, Bio‐TEM, JC‐10, and C11‐BODIPY assays were used. Bio‐TEM imaging showed that the normal mitochondrial structure of U87‐MG collapsed in **MHL@M** treated group compared with that of the control (Figure 4j). We further assessed mitochondrial membrane potential (MMP) using JC‐10 probe. As shown in Figure 4k, m, the strongest green fluorescence was observed in **MHL@M** group, indicating the potent disruption of MMP. Lipid peroxidation (LPO), a key indicator of ferroptosis, in U87‐MG cells was evaluated using the C11‐BODIPY probe. As shown in Figure 4l, after the administration of **MHL@M**, an increased green fluorescence signal was observed, indicating the increase of LPO, while the red fluorescence indicated that the unoxidized lipid decreased [47]. Quantitative analysis of the CLSM images further confirmed the enhanced LPO (Figure 4n). Notably, reduced cell viability, disrupted MMP and enhanced LPO were substantially reversed by Fer‐1, a commercially available ferroptosis inhibitor (Figure 4b, k–n). The results showed that **MHL@M** had an excellent tumor cell‐killing effect via the activation of ferroptosis (Figure 4o).

### 2.5 In Vivo Targeted Imaging and Anti‐Tumor Capacity of MHL@M

**FIGURE 5:**
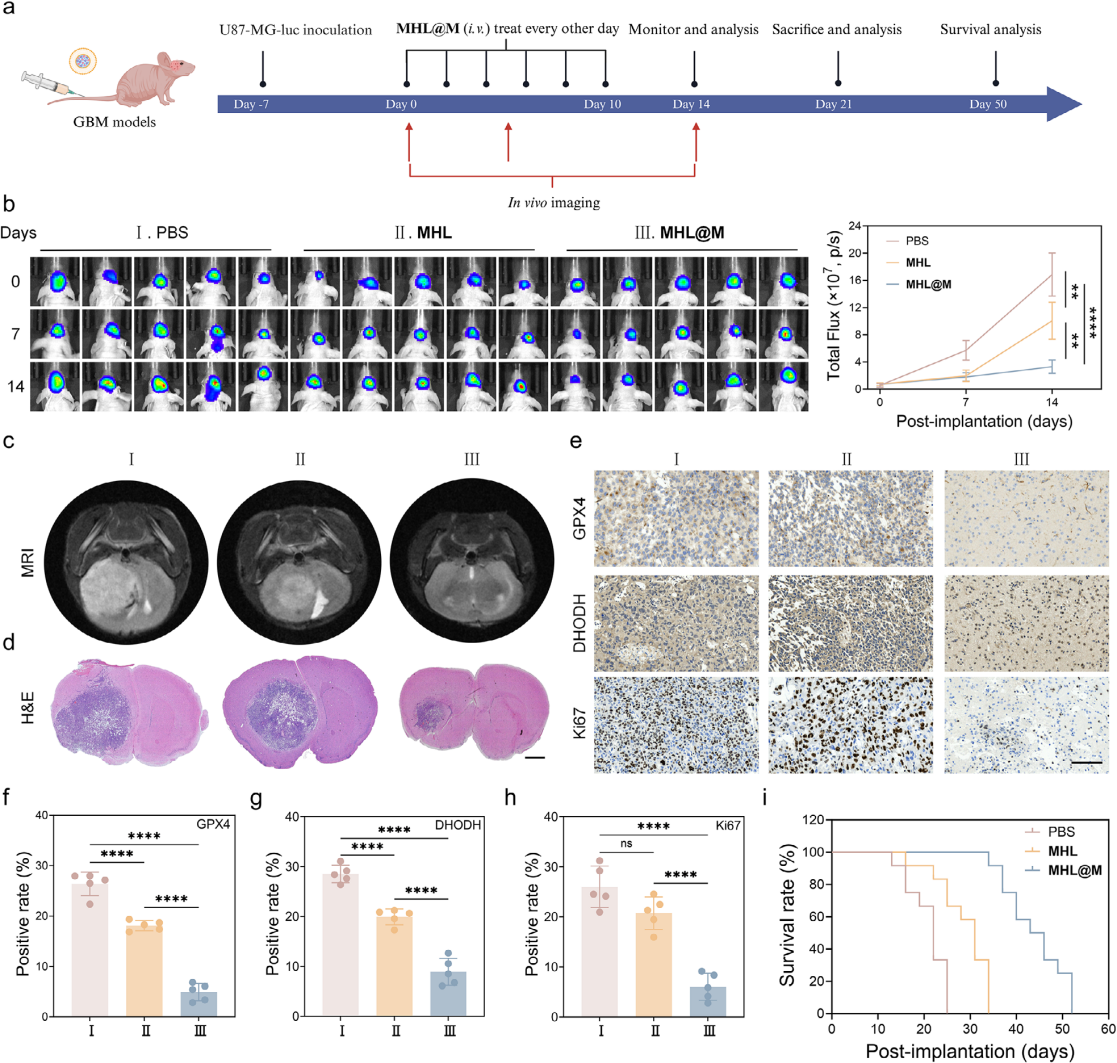
In vivo anti‐tumor performance of MHL@M. a) Treatment diagram of the experiment. b) Luminescence images of GBM‐bearing Balb/c nude mice treated with different treatments at the pre‐designed time points (*n* = 5). c) MRI transverse section view of GBM‐bearing mice at 14 days post U87 tumor cell inoculation (*n* = 5). d) H&E analysis of tumor tissues after administration of PBS, **MHL** and **MHL@M**. (*n* = 5, scale bar = 1 mm). e) IHC staining and f–h) corresponding quantitative analysis of tumor tissues in various groups (*n* = 5, scale bar = 100 µm). i) Kaplan‐Meier survival curves of the GBM‐bearing mice in different treatments (*n* = 5, I: PBS, II: **MHL**, III: **MHL@M**).

Based on the promising in vitro performance of **MHL@M**, we assessed its anti‐tumor activity in orthotopic GBM models (Figure 5a). We established an orthotopic GBM model by the intracranial injection of luciferase‐labeled U87‐MG (U87‐MG‐luc) cells into Balb/c nude mice. Tumor formation was confirmed with bioluminescence observed with an animal fluorescence imaging system (IVIS Lumina III, PerkinElmer) and magnetic resonance imaging (MRI, 7.0 T MRI system on a BRUKER BioSpec 70/20) on 14 days after orthotopic injection. As shown in Figure S20, GBM mice exhibited notable bioluminescence and MRI signals, confirming the successful establishment of an orthotopic GBM mice model. The tumor‐targeting ability of **MHL@M** was examined. As shown in Figure S21a, 4 h after intravenous administration of **MHL@M**, it was present in the brain of the orthotopic GBM model and the fluorescence signal reached peak at 10 h post‐injection. Even 24 h after administration, the fluorescence signal of **MHL@M** was observed in the brain; however, that of **MHL** was almost invisible, indicating the intracranial retention capacity of the U87‐MG CM coating (Figure S21b). Subsequently, the anti‐tumor effect of the **MHL@M** was investigated. **MHL@M** intravenous administration significantly inhibited GBM tumor growth, compared with that in the PBS or **MHL** administration groups, as demonstrated by the reduced bioluminescence signal (Figure 5b). We confirmed these anti‐tumor effects using MRI. As shown in Figure 5c, administration of **MHL@M** resulted in the smallest diameter of MRI signal, which aligned well with the bioluminescence imaging results. Hematoxylin and eosin (H&E) staining was also used to assess the anti‐tumor effect of **MHL@M**. Tumor volume in **MHL@M** treatment group was the smallest (Figure 5d). These results demonstrated the outstanding anti‐tumor capacity of **MHL@M**. Given the presence of hemin and leflunomide in **MHL@M**, we examined the expression of GPX4 and DHODH in tumors via IHC staining. This demonstrated that both GPX4 and DHODH signals were significantly downregulated by **MHL@M** compared with those in the PBS (Figure 5e–g). As shown in Figure 5e, h, the administration of **MHL@M** led to the weakest signal in the Ki67 staining assay which was widely used to reflect the cell proliferation rate. The above results demonstrated the excellent in vivo anti‐tumor property of **MHL@M**, which was established by inhibiting both GPX4 and DHODH and inducing ferroptosis.

Next, we assessed the biocompatibility of **MHL@M** by monitoring the body weight, survival rate, morphological structure of the main organs, and blood biochemical indicators. As shown in Figure S22, the administration of **MHL@M** alleviated the body weight loss induced by orthotopic U87‐MG transplantation. Moreover, the Kaplan‐Meier survival curve showed that **MHL@M** prolonged the survival time of tumor‐bearing mice (Figure 5i). H&E staining of the main organs showed no notable morphological alterations in the heart, liver, spleen, lung, and kidney, compared with those of the control (Figure S23). Additionally, serum alanine transaminase (ALT), aspartate transaminase (AST), blood urea nitrogen (BUN), and creatinine (CRE) level, the key biochemical parameters reflecting the function of liver and kidney, were measured with commercially available detection kits. The results showed that **MHL@M** administration did not substantially alter these parameters compared with those of PBS (Figure S24). To evaluate the long‐term biosafety of **MHL@M**, period of treatments was extended and Mn^2+^ was detected in the main organs to represent **MHL@M** metabolism. As demonstrated in Figure S25, Mn^2+^ was mainly in the liver and kidneys, where **MHL@M** was metabolized. These results indicated that **MHL@M** had excellent biosafety and substantial translational potential for clinical applications in GBM treatment.

### 2.6 The Anti‐Tumor Molecular Mechanism of MHL@M

**FIGURE 6:**
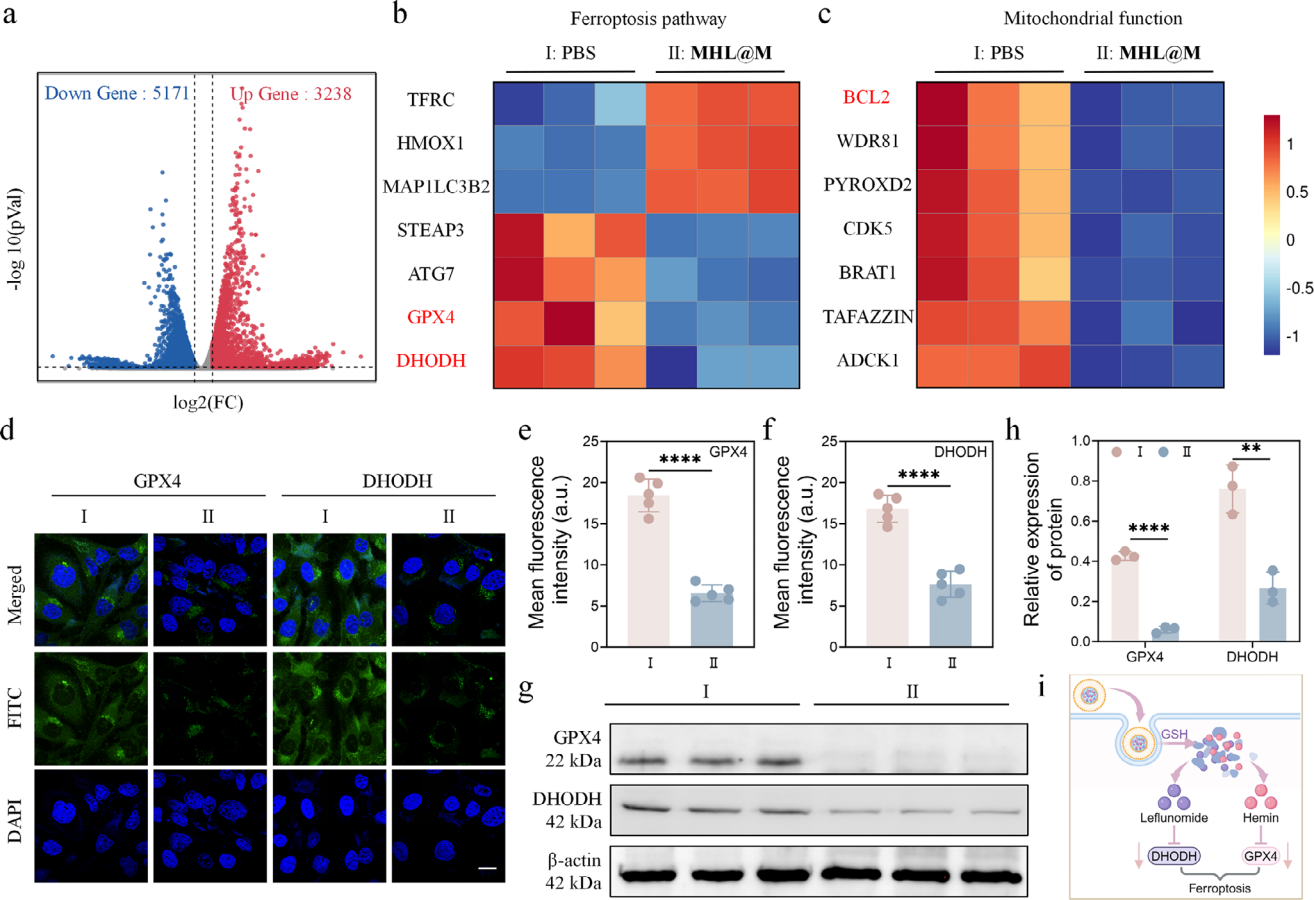
The anti‐tumor molecular mechanism of MHL@M. a) Volcano map of differentially expressed genes (DEGs) of U87‐MG cells treated with **MHL@M** for 24 h. b, c) Clustering heat map of DEGs. d) Confocal fluorescence images and e, f) quantitative analysis of the expression of GPX4 and DHODH in different groups (*n* = 5, scale bar = 20 µm). g) Western blotting analysis and h) quantitative analysis of the expression of GPX4 and DHODH in different groups (*n* = 3). i) Schematic diagram of the anti‐tumor mechanism of **MHL@M** (I: PBS, II: **MHL@M**).

Triggered by the excellent in vitro and in vivo anti‐tumor performance of **MHL@M**, we further explored the underlying molecular mechanisms using transcriptomics analysis. Principal component analysis (PCA) confirmed the credibility of the RNA sequencing data on **MHL@M** and PBS‐treated U87‐MG cells (Figure S26). The volcano plot showed that there were 8409 differentially expressed genes (DEGs) in U87‐MG cells treated with **MHL@M** compared to those treated with PBS. Among them, 5171 genes were downregulated and 3238 genes were upregulated (Figure 6a). The network diagram of Gene Ontology (GO) enrichment and Kyoto Encyclopedia of Genes and Genomes (KEGG) signaling pathways demonstrated that the significantly altered genes were mainly enriched in the cell ferroptosis processes (Figures S27–S30). Clustering heat map analysis further proved that bioprocesses, such as ferroptosis and mitochondrial function, were profoundly altered by **MHL@M** administration. Among them, GPX4, DHODH (the key mediators of ferroptosis), BCL2 (one classic anti‐mitochondria‐related cell death molecule) genes, were significantly downregulated, indicating the excellent pro‐ferroptosis effect of **MHL@M** (Figure 6b, c). To further verify the downregulation of GPX4 and DHODH observed in transcriptomic analysis, immunofluorescence (IF) and western blotting assays were performed. As shown in Figure 6d–h and Figure S31, after treatment with **MHL@M**, the expression of GPX4, DHODH, and BCL2 was significantly suppressed. These results indicated that **MHL@M** could induce ferroptosis and damage mitochondrial function by concurrently inhibiting both GPX4 and DHODH, thereby achieving an excellent anti‐tumor effect (Figure 6i).

## 3 Conclusions

Given the clinical challenges in GBM therapy, especially the overexpression of GPX4 and DHODH in GBM, we proposed and prepared a novel nanoplatform, **MHL@M**, to achieve the efficient GBM inhibition via provoking ferroptosis. **MHL@M** exhibited a high drug‐loading rate, and excellent GBM targeting, TME‐responsive, GSH‐depleting, and O_2_/ROS‐generation capacities. More importantly, **MHL@M** achieved an ideal anti‐tumor effect in vitro and in vivo by downregulating GPX4 and DHODH, which subsequently triggered ferroptosis. To be noted, several key aspects should be addressed to advance the clinical translation of **MHL@M**. First, its therapeutic performance should be validated in more clinically relevant models, such as patient‐derived xenograft models. Second, long‐term biosafety studies including assessments of potential chronic toxicity and immunogenicity, are essential to ensure clinical application. Finally, given the highly invasive nature of GBM, the efficacy of **MHL@M** against metastasis requires further investigation. Overall, this study contributed to the development of clinical intervention strategy using a biomimetic nanoplatform.

## 4 Experimental Section

Detailed experimental materials and methods can be found in the Supporting Information.

## Author Contributions

W.L., L.L., and C.Z. were responsible for initiating the project. G.R., X.W., and P.L. performed the **MHL@M** preparation, in vitro and in vivo experiments. H.W., Z.B., Q.G., B.M., Z.T., J.L., and F.F. contributed to the analysis and interpretation of the results and to the writing of the paper. W.L., L.L., and C.Z. supervised the entire project. All authors discussed the results and commented on the manuscript.

## Conflicts of Interest

The authors declare no conflicts of interest.

## Supporting information




**Supporting File**: advs74925‐sup‐0001‐SuppMat.docx.

## Data Availability

The data that support the findings of this study are available in the supplementary material of this article.
