# Supplementary material for: Biomimetic Nanoplatform for Targeted Glioblastoma Therapy via Concurrently Triggering GPX4/DHODH Mediated Ferroptosis
Source: Adv Sci (Weinh). 2026 Mar 23;13(31):e21236. doi: 10.1002/advs.202521236 (PMC13587953; doi:10.1002/advs.202521236)
Supplement: Supplementary file 1 [file ADVS-13-e21236-s001.docx]

Supporting Information

**Biomimetic Nanoplatform for Targeted Glioblastoma Therapy *via* Concurrently Triggering GPX4/DHODH Mediated Ferroptosis**

**Guodong Ren^#^, Xuewei Wang^#^, Panpan Li^#^, Hao Wu, Zhifei Bi, Qi Gao, Bolong Ma, Zixuan Tian, Jianfei Liu, Fuqiang Feng, Wen Liu*, Lin Li* & Chengwu Zhang***

**Dr. G. Ren, Ms. X. Wang, Mr. H. Wu, Ms. Z. Bi, Ms. Q. Gao, Ms. B. Ma, Ms. Z. Tian, Dr. J. Liu, Dr. W. Liu, Prof. C. Zhang**

**School of Basic Medical Sciences, Shanxi Medical University, Taiyuan 030001, China**

**Email:** [liuwen@sxmu.edu.cn](mailto:liuwen@sxmu.edu.cn;)**[;](mailto:liuwen@sxmu.edu.cn;)** [chengwu_zhang@sxmu.edu.cn](mailto:chengwu_zhang@sxmu.edu.cn)

**Dr. G. Ren, Ms. X. Wang, Ms. Z. Bi, Dr. J. Liu, Dr. W. Liu, Prof. C. Zhang**

**Shanxi Province Brain Degenerative Diseases Precision Diagnosis and Treatment Engineering Research Center, Shanxi Medical University, Jinzhong 030606, China**

**Mr. F. Feng**

**Department of Neurosurgery, Shanxi Province Cancer Hospital/Shanxi Hospital Affiliated to Cancer Hospital, Chinese Academy of Medical Sciences/Cancer Hospital Affiliated to Shanxi Medical University, Taiyuan 030013, China**

**Ms. P. Li, Prof. L. Li**

**State Key Laboratory of Flexible Electronics (LoFE) & Institute of Flexible Electronics (IFE), Northwestern Polytechnical University, Xi'an 710072, China**

**Prof. L. Li**

**State Key Laboratory of Flexible Electronics (LoFE) & Institute of Flexible Electronics (IFE), Xiamen University, Xiamen 361102, China**

**Email:** [ifelli@xmu.edu.cn](mailto:ifelli@xmu.edu.cn)

**^#^These authors contributed equally to this work.**

1. **Materials and methods**

**Reagents:** Hemin, Leflunomide, methyl thiazolyl tetrazolium (MTT), DMEM medium, fetal bovine serum (FBS), N-hydroxysuccinimide (NHS), 5,5-dithiobis-(2-nitrobenzoic acid) (DTNB), trypsin and calcein acetoxymethyl ester (calcein-AM) were purchased from Solarbio (Beijing, China). 2,7-dichlorodihydrofluorescein diacetate (DCFH-DA) and propidium iodide (PI) were obtained from Aladdin Co., Ltd. (Shanghai, China). Naphthalene-2,3-dicarboxaldehyde (NDA) and tetraethyl orthosilicate (TEOS) were obtained from MedChemExpress (Beijing, China). Anti-GPX4 antibody (Cat NO: A11243) was obtained from ABclonal Technology Co., Ltd. (Wuhan, China). Anti-DHODH antibody (Cat NO: 14877-1-AP), anti-BCL2 antibody (Cat NO: 12789-1-AP), anti-CD44 antibody (Cat NO: 15675-1-AP) and HRP-Goat Anti-Rabbit Recombinant Secondary Antibody (H+L) (Cat NO: RGAR001) were provided by Proteintech Co., Ltd. (Wuhan, China). Goat anti-rabbit IgG/FITC (Cat NO: bs-0295P-FITC) was supplied by Bioss Biotechnology Co., Ltd. (Beijing, China). All the reagents used in the experiments were analytical grade. All the experiments were performed by using ultra-pure water (≥18 MΩ‧cm).

**Apparatus:** The morphological characteristics of the nanomaterials were measured using an FEI Tecnai G2 20-S-TWIN transmission electron microscope (Hillsboro, OR, USA). Absorption spectra were recorded on a Mapada 760CRT spectrophotometer (Shanghai, China), while fluorescence spectra were acquired with a Varian Cary Eclipse fluorescence spectrophotometer (Palo Alto, CA, USA). Fourier transform infrared (FTIR) spectra were collected using a Varian FTIR-640 spectrometer (Palo Alto, CA, USA). Chemical bonding information of the nanomaterials was analyzed by X-ray photoelectron spectroscopy (XPS) on a Thermo Scientific ESCALAB 250Xi system (Waltham, MA, USA). Cellular uptake and treatment effects were evaluated using an Olympus FV-3000 laser scanning confocal microscope (Tokyo, Japan) and a BD FACS Calibur flow cytometer (New York, NJ, USA). For *in vivo* imaging, drug distribution was monitored with a Bruker Small Animal Optical Imaging System (Karlsruhe, Germany), and MRI was performed on a 7.0 T BRUKER BioSpec 70/20 instrument. The concentration of oxygen generated by **MHL@M** was measured with a dissolved oxygen meter (JPBJ-608, Leici, Shanghai, China).

**Clinical samples assay:** This project is approved by the Cancer Hospital Affiliated to Shanxi Medical University (Approval No. KY2025118). All participants provided written informed consent in accordance with institutional guidelines. The expression of GPX4 and DHODH in GBM patients were analyzed using the UALCAN and GEPIA database. To further validate their expression, IHC analysis was carried out on clinical samples (*n* = 5).

**Preparation of SiO_2_:** SiO_2_ was prepared via previous method.^[1]^ In detail, triton X-100 (5.3 mL), cyclohexane (22.5 mL), and *n*-hexanol (5.4 mL) were mixed and stirred for 5 min. Then, ammonia solution (0.75 mL) and deionized water (1 mL) were added, and stirred for another 30 min. Subsequently, a mixed solution containing tetraethyl orthosilicate (TEOS, 0.5 mL) and (3-aminopropyl)-triethoxysilane (APTES, 0.1 mL) were added, and reacted at room temperature for 24 h. The resulting mixture was centrifuged at 12,000 rpm for 10 min. The precipitate was collected and washed three times with mixture of ethanol and water (1 : 1). Finally, the purified precipitate was redispersed in water to obtain a silica suspension for further use.

**Fabrication of H-MnO_2_**: Potassium permanganate (100 mg) was dissolved and added to the silica suspension. Ultrasonic for 6 h, then stirred it at room temperature for another 12 h. Centrifuged (12,000 rpm, 10 min), and washed the precipitate three times with deionized water. The precipitate was resuspended in a 2 M sodium carbonate solution and incubated in an oil bath at 60℃ for 12 h to etch of the silica. The sample was then centrifuged (12,000 rpm, 10 min), and the resulting precipitate was washed three times. Finally, the product (H-MnO_2_) was lyophilized using a freeze dryer and stored for further use.

**Preparation of MHL:** To obtain multifunctional nanoplatform, H-MnO_2_, hemin, and leflunomide were mixed at a mass ratio of 1 : 3 : 3 and stirred at room temperature for 12 h. Centrifuged at 12,000 rpm for 10 min. The resulting precipitate was collected and washed three times with deionized water. Finally, the MnO_2_-Hemin-Leflunomide (**MHL**) was obtained via freeze-drying.

**Cell membrane extraction:** Human glioblastoma cells (U-87 MG) were purchased from Procell Life Science&Technology Co.,Ltd. (Cat NO: CL-0238, Wuhan, China). U-87 MG were seeded at a density of 1 × 10^6^ cells/dish. When the cells reached approximately 90% confluence, the culture medium was discarded, and the cells were washed three times with pre-cooled PBS to remove dead cells and metabolic waste. The cells were then scraped and subjected to two additional washes with PBS. Next, mixture of membrane protein extraction buffer A and pre-cooled PMSF, was added to the cell precipitant. Extraction was carried out on ice for 15 min, followed by centrifugation to collect the clarified lysate. The membrane fraction was subsequently obtained by high-speed centrifugation (14,000× g, 4°C, 30 min).

**Preparation of MHL@M:** The extracted U87-MG cell membrane was dissolved in water, mixed with **MHL (**1 : 1), and then processed with ultrasound and a mini-extruder in an ice bath to obtain **MHL@M**, which was stored at 4°C for future use.

**Drug loading efficiency and release kinetics of MHL@M:** To characterize the drug delivery performance of the synthesized **MHL@M** nanocomposites, we systematically determined their drug loading content (DLC) as well as the drug release behavior under simulated physiological conditions.

The DLC was quantified by the standard curve method, following the principle that unloaded free drugs remain in the supernatant after nanoplatform synthesis, while loaded drugs are immobilized in the **MHL@M** precipitate. In detail, after the preparation of **MHL@M,** the reaction mixture was centrifuged at 12,000 rpm for 10 min to completely separate the **MHL@M** precipitate from the supernatant containing unbound Hemin and Leflunomide. The absorbance of the collected supernatant was measured. The amount of unloaded drugs was calculated based on their pre-calibrated standard curves. DLC was then computed using the formula:

DLC(%) = (Initial drug amount−Unloaded drug amount)/Mass of **MHL@M**×100%

For the *in vitro* drug release assay, **MHL@M** dispersion (5 mg·mL⁻¹) was incubated with 10 mM glutathione (GSH) at room temperature for 24 h to mimic the intracellular reductive microenvironment. After incubation, the mixture was centrifuged at 12,000 rpm for 10 min, and the absorbance of the supernatant was measured. The amount of released drugs was calculated via the corresponding standard curves. The drug release efficiency was determined as the ratio of the released drug amount to the total loaded drug amount in **MHL@M**.

**Catalase-like and GSH-responsive capacities of MHL@M:** MnO_2_ was a well-established nanoplatform with catalase-like and glutathione (GSH)-responsive properties,^[2, 3]^ and it could be converted to Mn^2+^ which was one chemo-dynamic agent,^[4]^ implying that **MHL@M** had excellent O_2_/•OH-generation capabilities. The •OH generation performance was assessed *via* the degradation of methylene blue (MB). Briefly, a solution containing MB (10 μg∙mL⁻¹) and H_2_O_2_ (1 mM) was incubated with varying concentrations of **MHL@M** at 37°C for 30 min. The extent of •OH-induced degradation was quantified by measuring the decrease in absorbance of MB at 665 nm.

**Cell viability assay:** Cell viability was determined using the MTT assay to assess the cytotoxicity of drug-loaded nanocarriers at various concentrations. U87-MG and NHA cells in the logarithmic growth phase were seeded into 96-well plates at a density of 1 × 10^4^ cells/well. 24 h later, the medium was then replaced with 100 μL of fresh medium (control) or medium containing different concentrations of **MHL@M** (from 25 to 150 μg∙mL^-1^). After another 24 h, medium containing MTT (0.5 mg∙mL^-1^, 100 μL) was added to each well, and the plates were incubated at 37°C for 4 h. Then DMSO (150 μL) added and the absorbance at 490 nm of mixture was measured using a microplate reader. Then cell viability was calculated based on the optical density (OD) values.

**Cellular uptake:** Triggered by excellent *in vitro* performance, behavior of **MHL@M** at cellular level was further studied. Firstly, the cellular uptake efficiency of **MHL@M** was evaluated. U87-MG cells were seeded into confocal dishes at a density of 1 × 10^5^ cells/well and cultured for 24 h. Subsequently, 1 mL of **MHL@M** (100 μg∙mL⁻¹) loaded with Rhodamine B was added to each dish and incubated for different time. Then they were washed with PBS, and fluorescent imaging was carried out using confocal laser scanning microscopy (CLSM). Meanwhile, cellular uptake of **MHL@M** was quantitatively analyzed by flow cytometry.

**Blood-brain barrier penetration experiment:** U87-MG and mouse brain microvascular endothelial cells (bEnd.3 cells) served as model cells. bEnd.3 cells were seeded in the upper chamber of a transwell insert to form a monolayer simulating the blood-brain barrier (BBB), while U87-MG cells in the lower chamber mimicked the tumor environment. Transendothelial electrical resistance (TEER) was measured. And BBB model was successfully established when its values above 200 Ω∙cm^2^.^[5]^ Equal volumes of **MHL** and **MHL@M** loaded with Rhodamine B were added to the upper chamber. After incubation, fluorescent image was carried out.

**Tumor sphere penetration capacity of MHL@M:** The tumor tissue penetration capability of **MHL@M** was evaluated using a three-dimensional tumor spheroid model. Briefly, 96-well plates were pre-coated with 1.5% (w/v) agarose to prevent cell adhesion. U87-MG cells in the logarithmic growth phase were then seeded into the plates at a density of 2,000 cells/well and cultured. The medium was half-refreshed daily until the spheroids reached an appropriate size for the penetration assay. Subsequently, 100 μL of **MHL** and **MHL@M** loaded with rhodamine B (100 μg∙mL⁻¹) was added to each well and incubated for 24 h. After washing with PBS, the spheroids were visualized using CLSM.

**Detection of intracellular O_2_:** The catalase-like activity of **MHL@M** was further evaluated using [Ru(dpp)_3_]Cl_2_ (RDPP) as a probe whose fluorescence intensity decreases in presence of O_2_. Briefly, U87-MG cells were seeded in confocal dishes at a density of 1 × 10^5^ cells per well and cultured overnight. The cells were then treated with **MHL@M** (100 μg∙mL^-1^) for 24 h, followed by incubation with RDPP (10 μg∙mL^-1^) for 4 h. Fluorescent imaging was performed using CLSM.

**Establishment of orthotopic GBM models:** The *in vivo* experiments were approved by the Cancer Hospital Affiliated to Shanxi Medical University (Approval No. DWLL2025027). Five-week-old Balb/c nude male mice were purchased from Beijing HFK Bio-Technology Co., Ltd. (Cat NO: 13001A, Beijing, China) and acclimatized for over one week prior to experimentation. All animals were housed under standard conditions, and experimental procedures were performed in accordance with relevant guidelines. Before surgery, mice were fasted overnight with water deprivation. Following anesthesia, the cranial hair was removed, and each mouse was secured in a stereotaxic frame. The surgical site was disinfected with iodine, and a longitudinal incision was made through the skin and subcutaneous tissue. A cranial burr hole was drilled at the stereotaxic coordinates 1 mm posterior to the bregma and 2 mm lateral to the midline. A microinjection syringe was used to slowly inject 5 μL of a cell suspension containing 5 × 10^5^ U87-MG-luc cells vertically into the brain through the burr hole. After injection, the needle was withdrawn gently, and the hole was sealed with bone wax. The surgical area was rinsed with sterile PBS, and the incision was sutured intermittently followed by iodine disinfection. Postoperatively, mice were maintained in a warm environment until full recovery to prevent hypothermia.

**Evaluation of *in vivo* targeting capacity of MHL@M:** Following the establishment of orthotopic U87-MG GBM models, mice were randomized into two groups. The drugs (Rhodamine B-labeled **MHL** or **MHL@M**, 5 mg∙kg^-1^, 200 μL) were administered via tail vein injection. Subsequently, at designated time points (0 - 24 h), mice were anesthetized, and *in vivo* fluorescent imaging was performed. The average fluorescence intensity in the tumor area was recorded for quantitative analysis.

***In vivo* anti-tumor effect of MHL@M:** To evaluate the therapeutic efficacy of **MHL@M**, tumor volume measurements were conducted across different treatment groups.	Mice were randomly assigned into three groups: (Ⅰ) PBS; (Ⅱ) **MHL**; (Ⅲ) **MHL@M**. We compared baseline characteristics between groups using standardized differences to assess the success of randomization. The respective treatments were administered with different drugs *via* tail vein injection every other day. Body weights of the mice were recorded every three days throughout the experimental period.

***In vivo* biodistribution and pharmacokinetics evaluation:** After successful establishment of the tumor models, each mouse received a single tail vein injection of **MHL@M** (5 mg∙kg^-1^, 200 μL). To evaluate biodistribution, mice were euthanized at predetermined time intervals post-injection. Major organs were collected and weighed, and the concentration of Mn^2+^ (as a surrogate for **MHL@M**) in blood and tissues was quantified using inductively coupled plasma (ICP) analysis. The biodistribution was expressed as the percentage of injected dose per gram of tissue (% ID/g).

**Biocompatibility assessment of MHL@M:** After treatment, mice blood samples were obtained for evaluation of hepatic and renal functions. Key serum biomarkers were analyzed alanine transaminase (ALT), aspartate transaminase (AST), blood urea nitrogen (BUN), and creatinine (CRE). Major organs, including the heart, liver, spleen, lung, and kidney, were also collected and subjected to histological examination *via* haematoxylin-eosin (H&E) staining.

**Western Blot assay:** After various treatments, the cells were lysed on ice in RIPA buffer containing PMSF for 30 min, with vortexing once every 10 min. The lysate was centrifuged at 12000 rpm for 20 min at 4 ℃, and the resulting supernatant was collected. Protein concentrations were determined using a BCA Protein Assay kit. Equal amounts of total proteins were separated by 10% sodium dodecyl sulfate polyacrylamide gel electrophoresis (SDS-PAGE) and electro-transferred onto PVDF membrane. The membranes were then blocked with blocking buffer for 1 h at room temperature, followed by incubation with primary antibodies overnight at 4°C. After washing and shaking three times with Tween-20 (TBST), the PVDF membrane was then incubated with the corresponding secondary antibody at room temperature for 1 h followed by washing and shaking. Imaged with Bio-Rad Imaging System.

**Statistical analysis:** Quantitative results were expressed as the mean ± standard deviation. Data analysis was conducted with GraphPrism 8, utilizing a two-sided Student's t-test to evaluate differences. A *p*-value of less than 0.05 was considered statistically significant, with specific levels denoted as follows: **p* < 0.05, ***p* < 0.01, ****p* < 0.001, and *****p* < 0.0001. Randomization was applied to all sample/animal group assignments, and inferences were made within a 95% confidence interval. To ensure objectivity, all data acquisition and analyses were performed in a double-blind manner, with investigators unaware of the specific treatment of the samples.

2. **Supplementary figures**

**Figure S1.** Expression analysis of GPX4 in GBM using the UALCAN database.

**Figure S2.** Expression analysis of DHODH in GBM using the UALCAN database.

**Figure S3.** a) The correlation between the expression level of GPX4 and the malignancy as well as b) survival time of glioma were analyzed using the Chinese Glioma Genome Atlas (CGGA) database.

**Figure S4.** Standard curve plots of absorbance toward the concentration of a) hemin and b) leflunomide from 0 to 0.01 mg∙mL^-1^.

**Figure S5.** High-resolution XPS spectra of a) C1s, b) N1s, c) O1s, d) Mn, e) Fe and f) F results of **MHL**.

**Figure S6.** FTIR spectrum results of H-MnO_2_, hemin, leflunomide and **MHL**.

**Figure S7.** Release plots of a) hemin, b) leflunomide and c) Mn^2+^ from **MHL@M** under different physiological conditions .

**Figure S8.** a) TEM image of **MHL** incubated with GSH for 30 min. b) Zeta potential of H-MnO_2_ and **MHL** before and after incubation with GSH (*n* = 3, scale bar = 100 nm).

**Figure S9.** The absorbance spectra of **MHL@M**.

**Figure S10.** Western blotting analysis of the expression of CD44 in different groups. U87-MG cell (I), U87-MG membrane (II), **MHL** (III) and **MHL@M** (IV).

**Figure S11.** a) The DLS and b) Zeta potential of **MHL** and **MHL@M** (*n* = 3).

**Figure S12.** Hemolysis analysis of red blood cells in the presence of **MHL@M** with various concentrations. PBS and distilled water were used as negative and positive controls, respectively (*n* = 3).

**Figure S13.** a) Flow cytometry analysis of fluorescence intensity in U87-MG cells after incubating with **MHL@M** (100 μg∙mL^-1^) for different time. b) Flow cytometry analysis of fluorescence intensity in U87-MG cells after incubating with different nanoparticles for 4 h. c) Quantitative analysis of fluorescence intensity in U87-MG, MCF-7 and KYSE30 cells after incubation with **MHL@M** (*n* = 5). d) Flow cytometry analysis of fluorescence intensity in U87-MG, MCF-7 and KYSE30 cells after incubating with **MHL@M** (100 μg∙mL^-1^) for 4 h.

**Figure S14.** Statistically analyze the TEER values for 16 days (*n* = 3).

**Figure S15.** The viability of NHA cells incubated with different concentration of **MHL** and **MHL@M** for 24 h.

**Figure S16.** The viability of U87-MG cells incubated with different concentration of hemin and leflunomide for 24 h.

**Figure S17.** a) Colony formation experiment and b) quantitative analysis of U87-MG cells after different treatments (*n* = 3, Ⅰ: PBS, Ⅱ: **MHL**, Ⅲ: **MHL@M**).

**Figure S18.** a) Wound healing images and b) quantitative analysis of U87-MG cells treated with different treatments (*n* = 5, scale bar = 500 µm). c-e) Invasion and migration results of U87-MG cells treated with different treatments, (*n* = 3, Scale bar = 200 μm, Ⅰ: PBS, Ⅱ: **MHL**, Ⅲ: **MHL@M**).

**Figure S19.** a) Fluorescent imaging (scale bar = 100 µm), b) quantitative analysis and c) flow cytometry analysis of U87-MG cells treated with varying concentrations of **MHL@M** (0, 50 and 100 μg∙mL^-1^) using NDA as GSH indicator. d) Flow cytometry analysis of U87-MG cells treated with different groups using NDA as GSH indicator. e) Flow cytometry analysis of U87-MG cells treated with different groups using DCFH-DA as ROS probe (*n* = 5, Ⅰ: PBS, Ⅱ: **MHL**, Ⅲ: **MHL@M**).

**Figure S20.** a) Luminescent images of GBM-bearing Balb/c nude mice after U87-MG-luc administration (*n* = 5). b) MRI transverse section view of GBM-bearing mice at 14 days post U87-MG-luc cells inoculation.

**Figure S21.** a) Fluorescent imaging at pre-designed time points (0, 2, 4, 6, 8, 10, 12 and 24 h) after intravenous injection of **MHL** and **MHL@M**. b) *Ex vivo* fluorescence distribution in brain and major organs after intravenous injection of **MHL** and **MHL@M** (5 mg∙kg^-1^, 200 μL) at 10 h and 24 h.

**Figure S22.** Body-weight change curves of the GBM-bearing mice in different treatments (*n* = 5).

**Figure S23.** Representative H&E staining images of main organs (heart, liver, spleen, lung, and kidney) section of various treatment groups (*n* = 5, scale bar = 100 µm).

**Figure S24.** Serum biochemical indices a) ALT, b) AST, c) BUN and d) CRE of tumor-bearing mice after *i.v.* injection in different treatment groups for 21 days (*n* = 5, Ⅰ: PBS, Ⅱ: **MHL**, Ⅲ: **MHL@M**).

**Figure S25.** Metabolism plot of **MHL@M** in mice by measuring the concentration of Mn^2+^ in various tissues at different time points post injection. The concentration of Mn^2+^ was measured by ICP (*n* = 3).

**Figure S26.** Pearson correlation between **MHL@M** and PBS groups.

**Figure S27.** GO enrichment analysis of DEGs.

**Figure S28.** KEGG enrichment analysis of DEGs.

**Figure S29.** GO enrichment analysis of DEGs related pathways.

**Figure S30.** KEGG enrichment analysis of DEGs related pathways.

**Figure S31.** Confocal fluorescent images of the expression of BCL2 in different groups (*n* = 5, scale bar = 20 µm).

**References**

[1] H, Zhang, Y. B. Pan, Z. B. Wang, et al., “Synthesis of Hollow Mesoporous Manganese Dioxide Nanoadsorbents with Strong Negative Charge and Their Ultra-efficient Adsorption for Cationic Dyes,” *Separation and Purification Technology* 295 (2022): 121241.
[2] H. Li, X. Jin, B. Chu, et al., “Pro-inflammatory Endothelial Cell Activation Mediated by Protofibrillar Tau Leads To loss of Barrier Function and Integrity,” *Small* 21 (2025): e2500113.
[3] C. Li, C. Hua, C. Chu, et al., “A Photothermal-responsive Multi-enzyme Nanoprobe for ROS Amplification and Glutathione Depletion to Enhance Ferroptosis,” *Biosensors & Bioelectronics* 278 (2025): 117384.
[4] C. Yang, Y. Meng, Y. An, et al., “In Situ and Real-Time Multi-Modality Imaging Guided Orderly Triple-Therapy of Tumors with a Multifunctional Nanodrug,” *Advanced Sci*ence 12 (2025): e2501048.
[5] B. Han, W. Xie, Y. Zhang, et al., “The Influx/efflux Mechanisms of D-peptide Ligand of nAChRs Across the Blood-brain Barrier and Its Therapeutic Value in Treating Glioma,” *Journal of Controlled Release* 327 (2020): 384-396.
